# Confocal Raman microscopy to identify bacteria in oral subgingival biofilm models

**DOI:** 10.1371/journal.pone.0232912

**Published:** 2020-05-11

**Authors:** Lukas Simon Kriem, Kevin Wright, Renzo Alberto Ccahuana-Vasquez, Steffen Rupp

**Affiliations:** 1 Fraunhofer Institute for Interfacial Engineering and Biotechnology, Stuttgart, Germany; 2 Procter & Gamble, Egham, England, United Kingdom; 3 Procter & Gamble, Kronberg, Germany; University of Hong Kong, HONG KONG

## Abstract

The study of oral disease progression, in relation to the accumulation of subgingival biofilm in gingivitis and periodontitis is limited, due to either the ability to monitor plaque *in vitro*. When compared, optical spectroscopic techniques offer advantages over traditional destructive or biofilm staining approaches, making it a suitable alternative for the analysis and continued development of three-dimensional structures. In this work, we have developed a confocal Raman spectroscopy analysis approach towards *in vitro* subgingival plaque models. The main objective of this study was to develop a method for differentiating multiple oral subgingival bacterial species in planktonic and biofilm conditions, using confocal Raman microscopy. Five common subgingival bacteria (*Fusobacterium nucleatum*, *Streptococcus mutans*, *Veillonella dispar*, *Actinomyces naeslundii* and *Prevotella nigrescens*) were used and differentiated using a 2-way orthogonal Partial Least Square with Discriminant Analysis (O2PLS-DA) for the collected spectral data. In addition to planktonic growth, mono-species biofilms cultured using the ‘Zürich Model’ were also analyzed. The developed method was successfully used to predict planktonic and mono-species biofilm species in a cross validation setup. The results show differences in the presence and absence of chemical bands within the Raman spectra. The O2PLS-DA model was able to successfully predict 100% of all tested planktonic samples and 90% of all mono-species biofilm samples. Using this approach we have shown that Confocal Raman microscopy can analyse and predict the identity of planktonic and mono-species biofilm species, thus enabling its potential as a technique to map oral multi-species biofilm models.

## Introduction

Oral diseases, like gingivitis and periodontitis, are primarily caused by the accumulation of dental biofilm in the subgingival region [1,2]. Socransky et al. defined a model which explained the inter-relationship of bacterial species within an subgingival biofilm, associating specific organisms with health and disease status and placing these consortia into ‘Socransky’s complexes’ [3]. Examples of the Socransky complexes described that prevalence of *A*. *naeslundii* in the microbiota of a healthy periodontal region, whereas the prevalence of *Veillonella* was more associated with the plaque present in periodontitis. Abusleme et al. also identified subgingival species in periodontal healthy patients and patients with periodontitis [2].

Subgingival biofilms consist of a variety of species in nature, with a different composition and abundance of species found in varied locations within the biofilm [4,5]. It has been demonstrated that microorganisms cluster within a subgingival biofilm creating specific ‘hotspots’ of high bacterial densities of certain species [6,7].

Although biofilm architecture and dynamics are well understood, there is only a limited amount of research on the composition and development of bacteria within these biofilms using spectroscopic analysis techniques.

To this day, the most common technique to display the architecture of oral biofilms is the use of fluorescence *in-situ* hybridization (FISH) combined with confocal laser scanning microscopy (CLSM) [8–10]. While this technique shows good resolution and characterization within a biofilm, the main limitations are the complex preparation procedure, the cost and time associated with these measurements [11].

Optical spectroscopy techniques nowadays offer the opportunity to identify chemical compounds in high spectral resolution [12], combining the power of 3D sample analysis with focused chemical composition. One of these techniques is Confocal Raman Microscopy (CRM) that utilizes a laser beam with known wavelength to analyze a sample. By measuring the scattered radiation and energy shift, Raman is able to use the acquired information of a cell’s chemical characteristics to differentiate species [13]. This should allow for a quick, affordable and unaltered evaluation of biofilm samples with a spatial resolution of 1 μm.

Previously, CRM has been successful in the spatial resolution of biomedical environments like tissue samples or bacterial cells [14–18]. However, the acquired signals from these biomedical components are highly complex [19–21]. Therefore, differentiation based on Raman spectra has significant limitations so far [16,22–25]. To the best of our knowledge, only a few studies employed the use of CRM for environmental biofilms [26,27], but there has been very limited information in the field of oral biofilm mapping [23,28]. While surface enhanced Raman scattering (SERS) remains promising in combination with Raman microscopy [13,29,30] with the potential for higher levels of discrimination spectra, this study focuses on the analysis of chemically un-modified biofilm samples. The bacteria used for our studies were selected considering Socransky’s complexes and Abusleme’s analysis in the evaluation of subgingival biofilm species [2,3].

Because bacteria in subgingival biofilms live in the same habitat, they show similar chemical compositions. While differences in spectral fingerprint patterns of oral bacteria have been shown to be minor, they still allow differentiation between species to be made [22,31]. Given these differences it is therefore important to apply statistical models to spectral data to discriminate between species. Some of the major statistical approaches for spectral analysis are Principle Component Analysis (PCA) and multiple variations of Partial Least Square (PLS) analysis. In analytical statistics, PCA treats all variables in a database the same and uses high-dimensional points for classification but does not consider assigned classifications of variables [14,18,20,32–34]. In comparison, PLS uses these annotated classifications to maximize inter-class variance [35–38]. For that reason, PCA is normally used for simple and linear dimensionality reduction while PLS is used for classification of different sample groups.

In this research, we hypothesize that Raman spectroscopy coupled with prediction models can differentiate common oral bacteria from several different Socransky complexes which are also part of the core subgingival microbiome described by Abusleme et al (*Fusobacterium nucleatum*, *Streptococcus mutans*, *Veillonella dispar*, *Actinomyces naeslundii* and *Prevotella nigrescens*) [2,3]. We have used planktonically grown microbes to develop reference spectra and then used these to interrogate the identity of both planktonically and biofilm grown organisms. Combining the model with multivariate analysis can provide the opportunity to perform biofilm mapping with a non-invasive, high resolution approach. Due to resolution limitations of 1μm of the instrument’s laser beam it may not be possible to differentiate single cells in a biofilm model. However, ‘hotspots’ of high mono-species densities of size bigger than the detection limit should allow identification of species in multi-species biofilm models in the future. The purpose of this study is to lay the groundwork for using CRM in *in-vitro* research for subgingival biofilm models with the analysis of different oral subgingival species and its application to artificial subgingival mono-species biofilm models.

## Materials and methods

### Planktonic sample preparation

Laboratory stocks of *S*.*mutans* (ATCC35668), *A*.*naelsundii* (ATCC12104), *V*.*dispar* (ATCC 17748), *F*.*nucleatum* (ATCC 25586) and *P*.*nigrescens* (ATCC33563) were cultured in Falcon tubes with Brain Heart Infusion Medium (Sigma-Aldrich) and incubated under anaerobic conditions (80% N_2_, 15% CO_2_, 5% H_2_). Each bacterial stock was diluted in fresh media, separated into three samples that were inoculated for a total of 96h.

For calibration spectra, every 24h 1,5 mL of the sample was put in Eppendorf tubes and was centrifuged at 5000rpm for 5min. After centrifugation, the supernatant was removed. Samples were then resuspended in saline (0.9% NaCl w/v) and centrifuged for an additional 5min. After centrifugation, the supernatant was removed and the bacterial pellet was spread on a borosilicate glass slide (VWR) for confocal Raman spectral analysis. For the calibration spectra, the same experimental and total of nine samples per organisms were used.

### Biofilm sample cultivation

Mono-species biofilms were grown on CDC reactor glass coupons (Biosurface Technologies Corporation, Bozeman, MN, USA) in 24-well polystyrene cell culture plates (Nunc A/S, Roskilde, Denmark) using similar materials and methods of biofilm formation presented elsewhere [39]. Wells with glass coupons were filled with a mixture of PBS at pH 7.2 (800μL), modified fluid universal medium (mFUM, 800μL) and had a final glucose concentration of 0.15% (w/v) [39,40]. Wells were inoculated with bacterial species (200 μL) and incubated anaerobically at 37°C. The medium was renewed after 17h and 41h and glass coupons were dip washed three times in saline solution (0.9% NaCl w/v) after 17h, 25h, 41h and 49h to simulate saliva flow and to remove planktonic cells from the coupons. Biofilm coupons were removed from the wells after 65h, dip-washed in a saline (0.9% NaCl w/v) solution three times, and placed on glass slides (VWR) to be dried at 50°C for 30min.

### Instrumentation and data acquisition

The instrument used for analysis was a ThermoFisher Scientific DXR2xi, which was equipped with a 50x long working distance objective, a 532nm filter and a 532nm laser to capture a full spectral range of 50-3500cm^-1^. Data acquisition was performed using a 25μm confocal pinhole setup, 5.0mW laser power, 0.25s exposure time, 100 scans, a low baseline correction and a spectral detection range from 600-1800cm^-1^, also referred to the ‘fingerprint region’. For every spread sample of planktonic cells four random points were chosen on the bacteria-coated glass slide for spectra to be taken, resulting in 12 samples for every time point. For the biofilm samples, five random points were selected and spectra were acquired from six coupons for every species.

### Data analysis

Spectra were pre-corrected using low baseline correction of the ThermoFisher Scientific DXR2xi instrument. All spectral analyses and reprocessing were performed using Renishaw WiRE 5.2 (Renishaw plc, Wotton-under-Edge, UK). Each spectrum was pre-processed in the same way to reduce noise effects and spectral variations due to spectral sample collection. First, cosmic ray removal and baseline subtraction using intelligent polynomial algorithms was used to delete baseline noise from the borosilicate background. Then, noise filtering was applied taking into consideration all spectra from one organism. To additionally reduce noise, a 10 point Savitzky-Golay algorithm was applied to the spectra. All spectra were then normalized on a scale from 0 to 1.

After data acquisition and processing, the planktonic bacterial spectra data were divided into two groups–a calibration group for every species (21 samples with the selection of four random point spectra, 84 spectra) and a validation group (9 samples with the selection of 4 random point spectra, 36 spectra). Six samples with the selection of five random point spectra were chosen for the prediction of biofilm species.

The SIMCA Analysis (Umetrics, Umea, Sweden) was used for statistical proof. Two-Way Orthogonal Partial Least Square with Discriminant Analysis (O2PLS-DA) of the first derivative of the spectra was applied to differentiate species and cross-validate the model. O2PLS-DA is an analysis technique that allows differentiate systematic variation by correction their orthogonal variation in the X and Y matrices. The technique then decomposes the X and Y matrices into a joint, orthogonal and noise system. By using these systems, discriminant analysis is then able to consider and differentiate species based on their unique characteristics, discriminating the bacterial spectra into a two-dimensional score plot.

## Results

### Bacterial spectra for five different oral species

**Fig 1:**
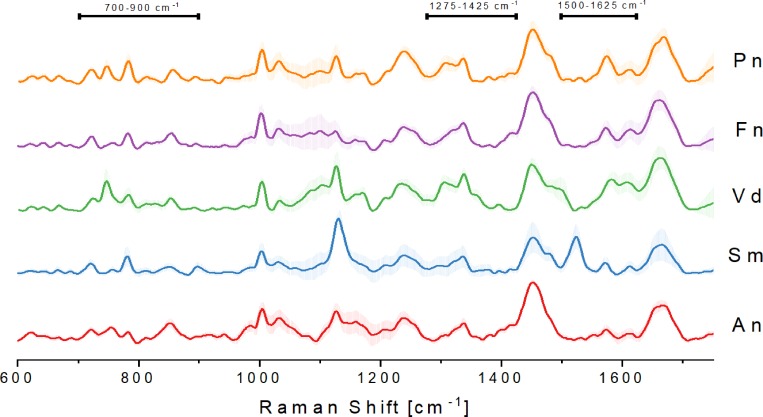
Averaged processed Raman signal (84 spectra total) from five different subgingival species of the calibration group with standard deviations. *A*.*naeslundii* (An), *S*.*mutans* (Sm), *V*.*dispar* (Vd), *F*.*nucleatum* (Fn) and *P*.*nigrescens* (Pn). Areas of differences in bands between species are indicated (700–900 cm^-1^, 1275–1425 cm^-1^, 1500–1625 cm^-1^).

Spectra of five different bacterial strains were analyzed using CRM as described in Materials and Methods. Fig 1 shows the plots of averaged Raman spectra (a total of 84 spectra per strain). While many vibrational bands were similar in the acquired spectra, several unique bands could be assigned to each of the individual bacterial species (S1 Table) that have been assigned previously. By selecting these unique band patterns, it is possible to discriminate between the individual species. Using a reference database of Raman spectra, it was previously shown that it is possible to identify predominant chemical signature patterns in similar and distinct spectral bands [18,41,42]. The presence of proteins is indicated by Amide I and Amide III bands that are most significant at ~1250 cm^-1^ (Amide III) and ~1660 cm^-1^ (Amide I). Amino acids are identified as Phenylalanine at ~1000 cm^-1^ and C-N and C-C stretches (specific for proteins) are found at ~1125 cm^-1^. CH_2_ deformations at ~1450 cm^-1^ are the result of lipids in the cell. Our results for *Streptococci* species are identical with the results from Berger *et al*. [31], who previously identified components in *S*.*mutans* and *S*.*sanguinis* (Amide I at 1651 cm^-1^, C-H_2_ deformation at 1457 cm^-1^, C-N and C-C stretch at 1127 cm^-1^, phenylalanine at 1005 cm^-1^; Raman shift can appear due to the use of a different Raman analysis setup).

Differences in band patterns between the five selected species are found mostly in the region between 700–900 cm^-1^. This area is specific for nucleotides (DNA and RNA) due to ring breathing vibrations. Additionally, the area between 1500 and 1625 cm^-1^ shows bands that are the results of different amino acid compositions within bacterial species and thus can be used for differentiation of species.

### Multivariate analysis of spectra

**Fig 2:**
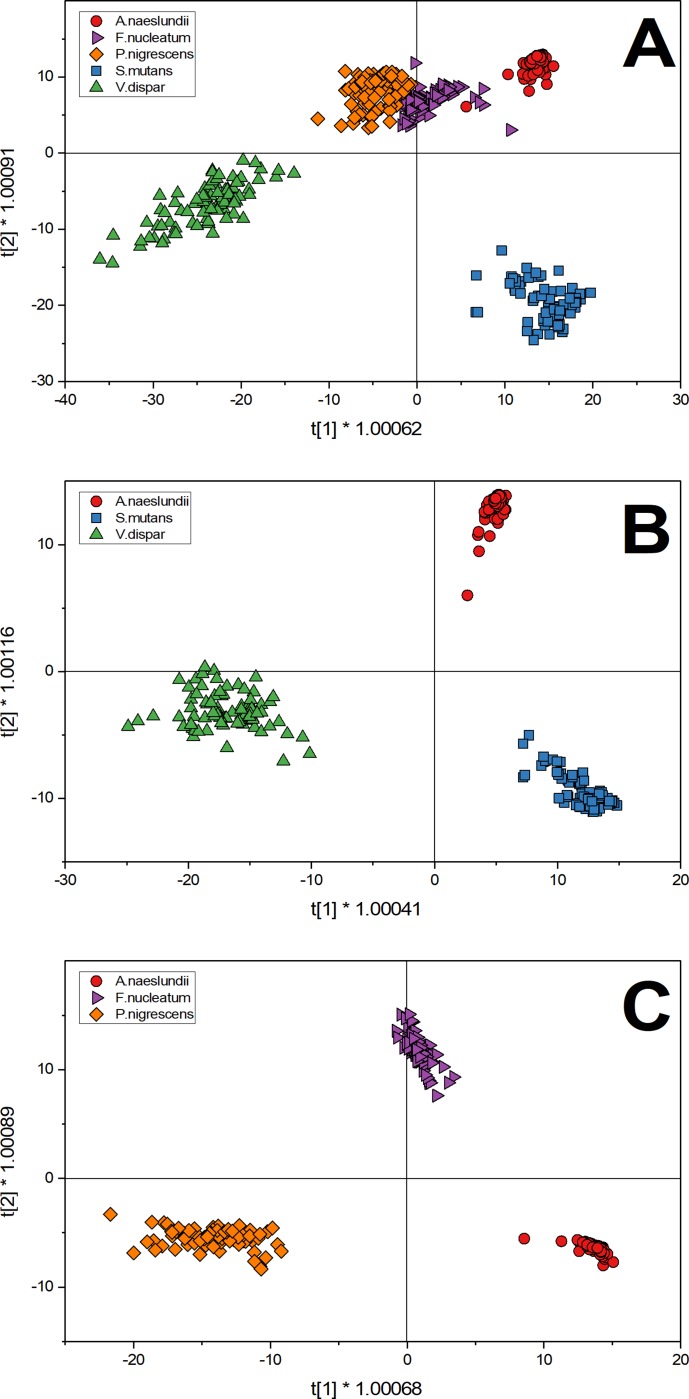
O2PLS-DA analysis of selected oral bacteria show the distribution of the first derivative of spectra (84 spectral samples for each strain) after discriminant analysis in a score plot. A) T-score plot of five species: *A*.*naeslundii* (red circles), *S*.*mutans* (blue square), *V*.*dispar* (green triangle), *F*.*nucleatum* (purple triangle) and *P*.*nigrescens* (orange diamond). B) T-score plot of three species that showed distinct clusters in the five species score plot: *A*.*naeslundii* (red circles), *S*.*mutans* (blue square) and *V*.*dispar* (green triangle). C) T-score plot of three species that showed one cluster in the five species score plot: *A*.*naeslundii* (red circles), *F*.*nucleatum* (purple triangle) and *P*.*nigrescens* (orange diamond).

O2PLS-DA was used as an analytical technique for the characterization of complex bacterial spectra shown in Fig 1. As described above, Spectra were acquired for five individual oral biofilm species (84 spectra each). Fig 2 shows T-score plots of different scenarios considering the two greatest variations in the datasets and plotted as X and Y.

Fig 2A shows a plot summary of the results of the O2PLS-DA of the five species. According to the O2PLS-DA algorithm, every data point shown represents a Raman spectra and also contains information of all measured species. Based on the selection of the two greatest variances, three clearly distinct clusters can be identified while one cluster shows an overlap of three species (*A*.*naeslundii*, *F*.*nucleatum*, *P*.*nigrescens*). Spectra for *V*.*dispar* can be found exclusively in the third quadrant while *S*.*mutans* are found in the fourth. Considering the third cluster, *P*.*nigrescens* is found only in the second quadrant and *A*.*naeslundii* in the first quadrant. Only *F*.*nucleatum* shows distributions in two quadrants (first and second). Since variance of samples are based on the average of a specific dataset Fig 2A considers all five species to build the average which results in insufficient separation of *A*.*naeslundii*, *F*.*nucleatum* and *P*.*nigrescens* because their calculated variances and thus Raman spectra are too similar.

In order to further analyze the data, Fig 2B was restricted to the analysis of the three most distinct species in the initial score plot. Based on the calculated variances three clearly separated clusters can be found in the same clusters and quadrants as in Fig 2A. Here, data points are spread less across the t[1]-axis than in Fig 2B but separation of species remains clear in Fig 2A. By further focusing on the analysis of data, Fig 2C shows the O2PLS-DA T-score plot of the three species that could not be differentiated in the initial five species T-score plot. Here, the three overlapping species from Fig 2A could be more clearly discriminated because the analysis now considers the average of the spectra of *A*.*naeslundii*, *F*.*nucleatum* and *P*.*nigrescens* for the calculation of variation. This change allows the separation of species because their two greatest variations change due to a recalculation of their average.

In order to set up an appropriate calibration dataset it is necessary to select species that show the highest degree of variance. In this work we selected *A*.*naeslundii*, *V*.*dispar* and *S*.*mutans* to set up our calibration dataset.

### Prediction of planktonic and mono-species biofilm cells

In a second step, species predictions of known samples were made. The acquired spectra from Fig 2B for planktonically grown microbes, were used to create calibration datasets for every individual species. This dataset was then used for prediction of mono-species planktonic and biofilm spectra using cross validation with the O2PLS-DA model. As a result of this prediction, spectra were associated to one of the three strains based on their similarities of spectral patterns to the calibration set. Two sets of predictive analysis were carried out using this training data set: 1. predict the identity of planktonically grown organisms and 2. predict the identity of biofilm grown organisms.

#### Prediction of unknown, planktonically grown isolates

**Fig 3:**
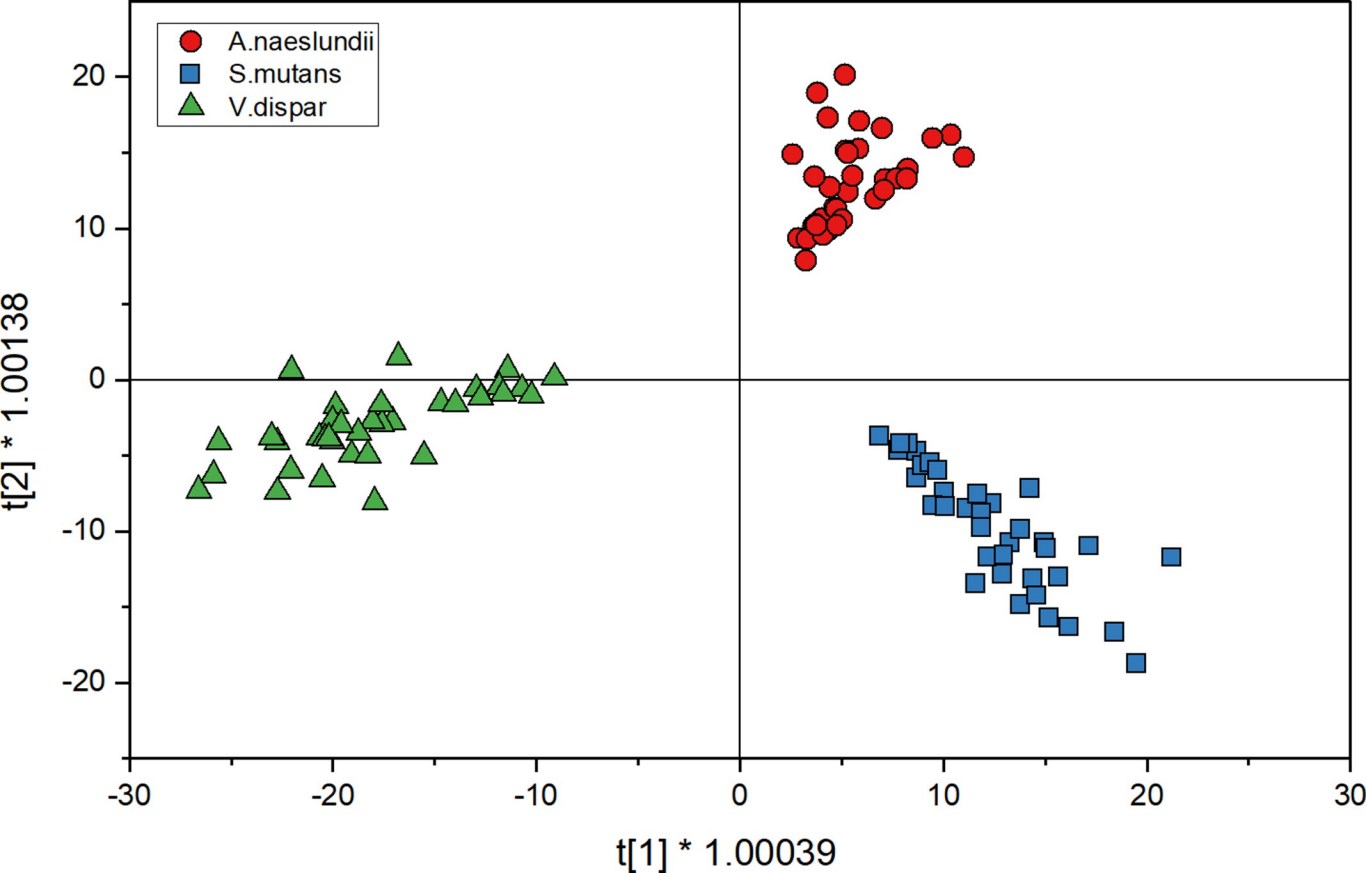
O2PLS-DA analysis of planktonic cell spectra for three species shows the distribution of spectra (36 spectra for each strain) after discriminant analysis in a 2D sphere; *A.naeslundii* (red circles), *S.mutans* (blue squares) and *V.dispar* (green triangles).

Fig 3 shows the score plot for planktonic cell spectra for three species when analyzed with O2PLS-DA. The distribution in each of the quadrants is the same as the calibration spectra but the spectra are more spread in the score plot. After the spectra were collected for prediction, they were compared to the model that has been set up with calibration spectra (Fig 2B) for cross validation. Table 1 shows the comparison of predicting planktonic cells using the O2PLS-DA model. The diagonal, bold values show the spectra that agree with the calibration spectra while the other values represent the errors of classification. Here, none of the spectra we misclassified by the model which results in a prediction accuracy of 100% for a total of 36 spectra for each strain.

**Table 1:** Comparison of the performance of species identification using the O2PLS-DA model for planktonic cells. The columns indicate the known/calibration species; the rows indicate the prevalence of predicted species using the O2PLS-DA model of the known/calibrated species spectra.

	Known Species		
	*A*.*naeslundii*	*S*.*mutans*	*V*.*dispar*
Predicted			
*A*.*naeslundii*	**36 (100%)**	0	0
*S*.*mutans*	0	**36 (100%)**	0
*V*.*dispar*	0	0	**36 (100%)**
Total Successful Prediction:	108 (100%)		

#### Predicting the identity of biofilm grown isolates

**Fig 4:**
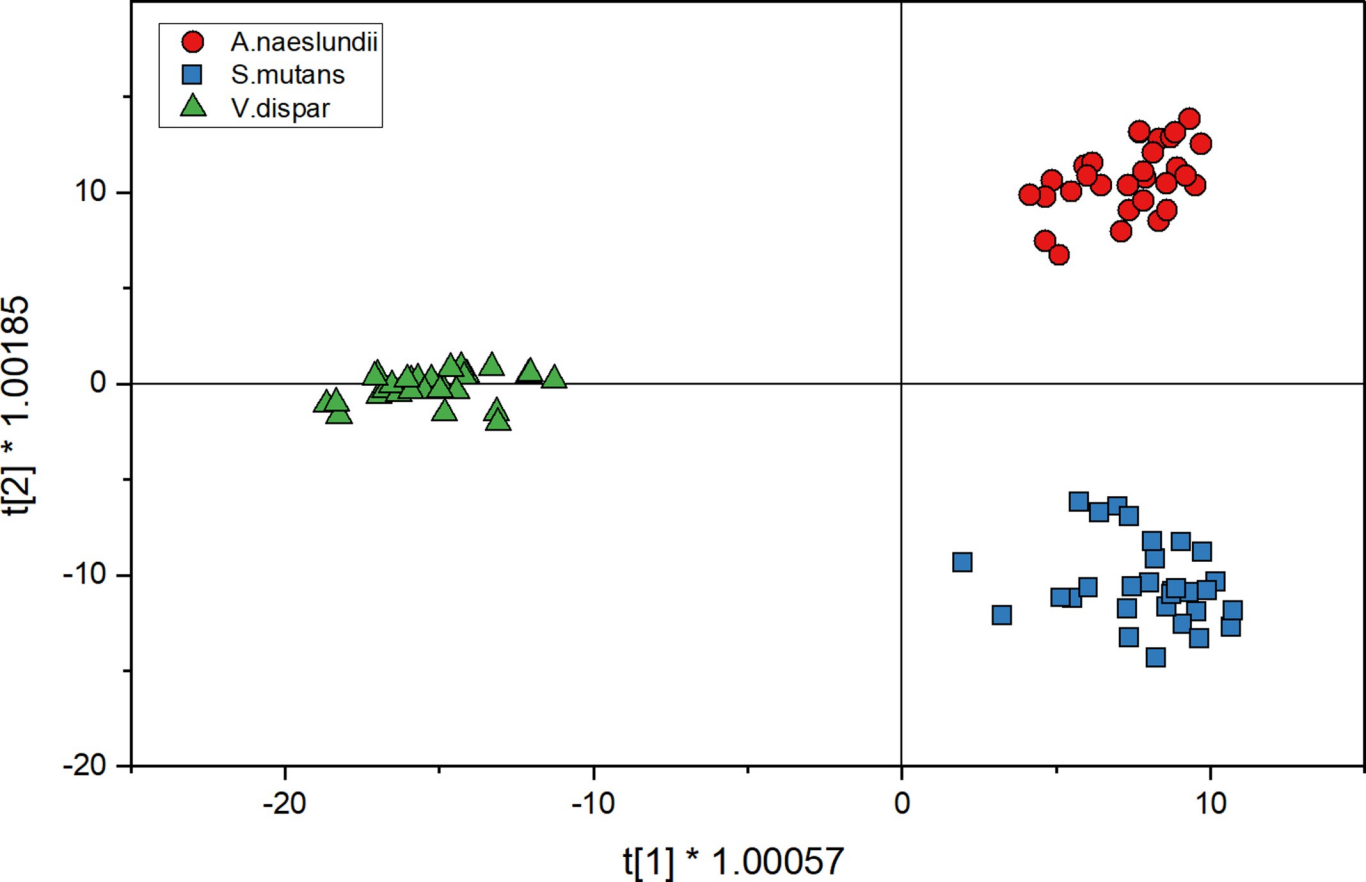
O2PLS-DA analysis of mono-species biofilm spectra (30 spectra for each strain) for three species shows the distribution of spectra after discriminant analysis in a 2D sphere; *A.naeslundii* (red circles), *S.mutans* (blue squares) and *V.dispar* (green triangles).

Fig 4 shows the score plot for mono-species biofilm cell spectra for three species when analyzed with O2PLS-DA. The distribution into quadrants is the same as for the calibration spectra. In Fig 4, the *V*.dispar cluster was distributed between the second and third quadrant. In comparison to the O2PLS-DA plot from the planktonic spectra, clusters are more defined here and closer together. For the prediction of species, spectra were also compared to the model that has been set up with calibration spectra (Fig 2B) for cross validation of the model. Table 2 shows the comparison of predicting mono-species biofilm cells using the O2PLS-DA model. While using the training set of data enabled the correct identification of more than 76% if *S*.*mutans*, more than 90% of *V*.*dispar* and 100% of *A*.*naeslundii*, the approach seems less sensitive from a prediction point of view.

**Table 2:** Comparison of the performance of species identification using the O2PLS-DA model for mono-species biofilm spectra. The columns indicate the known/calibration species; the rows indicate the prevalence of predicted species using the O2PLS-DA model of the known/calibrated species spectra.

	Known Species		
	*A*.*naeslundii*	*S*.*mutans*	*V*.*dispar*
Predicted			
*A*.*naeslundii*	**30 (100%)**	0	0
*S*.*mutans*	2 (6.6%)	**23 (76.7%)**	5 (16.7%)
*V*.*dispar*	0	2 (6.7%)	**28 (93.3%)**
Total Successful Prediction:	81 (90%)		

## Discussion

In this study we developed CRM methods, including data analysis tools to identify individual bacterial species in mono-species biofilm models. Due to their similar genetic and metabolic characteristics, the chemical composition in a bacterial cell between the tested subgingival oral bacteria showed limited but unique differences in their Raman-spectra. However, using O2PLS-DA we were able to distinguish several species. Similar results were obtained for *Uncaria* species in a different application as described in Feng *et al*. [35] when O2PLS-DA was applied. We were able to identify *S*.*mutans* and *V*.*dispar* and *A*.*naeslundii* grown in suspension and in mono-species biofilms after establishing a calibration set of spectra generated from planktonically grown cells, both in cells derived from planktonic cells and from biofilms [3]. The three species examined were found to coincide within the different complex groups described by Socransky, indicating significant chemotypic differences between these species. *P*.*nigrescens* and *F*.*nucleatum*, on the other hand, can be found within the same Socransky complex, indicating a close relationship, and consequently show substantial overlap in Raman spectra.

When performing O2PLS-DA with three selected species from three different Socransky complexes, it was possible to reliably identify spectral differences enabling species differentiation. Additionally, *A*.*naeslundii*, *S*.*mutans* and *V*.*dispar* could already be distinguished within the pool of five species. Thus, these three species were chosen for prediction of planktonic and mono-species biofilm cells.

Comparing the prediction score plots for both, planktonic and biofilm spectra, we observed that the clusters generated from planktonic cells is more spread out while the cluster for biofilm spectra remains compact with little distribution in the plot. This could be the result of bigger variance in the planktonic cells, resulting in a higher variance in signals between spectra, which broadens the cluster. Biofilm spectra on the other hand show less variance, indicating that the collected biofilm cells have more uniform appearance in Raman spectra.

Nevertheless, we could predict the species *A*.*naeslundii*, *S*.*mutans* and *V*.*dispar* with an accuracy of 100% if grown planktonically. For biofilm cells we achieved an accuracy of 90%. Because biofilm cells show changes in spectra if compared to planktonic cells (see S1 Fig), prediction of biofilm cells using data from planktonic cells for calibration might be problematic. This might be the reason for increased misclassification of biofilm cells, indicating the need for a biofilm based dataset for calibration. The change in Raman signal for biofilm cells is most likely the result of a change in metabolism when transitioning from a planktonic to a biofilm state [43].

Even though a total accuracy of 90% for biofilm cells was achieved, especially *S*.*mutans* shows low predictions accuracy (76.7%) compared to *A*.*naeslundii* (100%) and *V*.*dispar* (93.3%). *S*.*mutans* is known to be a key contributor in the production of exopolysaccharides (EPS) while *A*.*naeslundii* and *V*.*dispar* are lacking the ability to form EPS when grown as mono-species biofilms. Hence, low prediction accuracy can be the result of spectral interferences from EPS in *S*.*mutans* biofilm and needs to be considered in future experiments [44–46].

One of the most significant changes in the spectra is the absence of the 1525 cm^-1^ band in *S*.*mutans* representing increased amounts of polypeptides in planktonic cells. Due to the absence of this specific band, differences between *V*.*dispar* and *S*.*mutans* become much smaller which leads to several misclassifications of these biofilm spectra. For future experiments, it may be necessary to consider using biofilm spectra for the calibration O2PLS-DA model because of the change of band signal between planktonic and biofilm spectra.

High similarities in Raman spectra can be observed for species within the same Socransky complexes. Additionally, using CRM with higher resolution might improve this limitation in future experiments.

With this work, we tried to lay the groundwork whether differentiation of subgingival bacteria was possible using CRM and to determine if this technique can be applied for 3D biofilm modelling in the future. We were able to demonstrate that it is possible to differentiate oral bacterial species based on their Raman spectra using an O2PLS-DA model. It was possible to predict 100% of planktonic cells and 90% of biofilm cells from three species of three Socransky clusters. This indicates the possibility to differentiate bacterial clusters with different prevalence in orals diseases like periodontitis and gingivitis. In future research, it will be pivotal to apply this model not only to mono-species subgingival biofilm models but also to assess multi-species biofilm models. As part of this this development it will be necessary to evaluate the limitations of the technique and how this method behaves in saliva based biofilm models. While it may not be possible to differentiate all species present, species with higher abundances may be differentiated in a 3D structure successfully.

While the specific experiments showed the discrimination of planktonic bacteria and the prediction of mono-species biofilms, the method presented can be applied to build a spectral Raman library of subgingival bacteria. This can make it easier to assess and determine spatial distribution of artificial multi-species biofilm models in the future. For the analysis of mono-species biofilm models the established method was able to predict species successfully. However, in a multi-species setup it is necessary to validate and evaluate results from CRM with other methods like quantitative polymer chain reaction or CLSM. Due to the nature of sample preparation for CLSM and the increased fluorescent signal it will be essential to use coherent anti-stokes Raman scattering to omit the signal from FISH in the spectral analysis.

## Conclusions

In the present study, confocal Raman microscopy coupled with two-way orthogonal Partial Least Square with Discriminant Analysis was applied to 1) discriminate between three oral bacterial species and 2) to develop a prediction model to successfully predict species planktonic cells and cells in a mono-species biofilm model. It was possible to identify 100% of planktonic spectra and 90% of mono-species biofilm spectra correctly. Future work will include the application of the model to the discrimination of oral bacteria in a multi-species biofilm. The developed method should allow spatial prediction of species using the fingerprint region (600-1800cm^-1^) of Raman spectra. It is pertinent to note that in order to perform special predictions (in two or three dimensions) it is necessary to combine batch spectra processing with the developed multivariate analysis technique in a workflow to use multivariate analysis for multi-species biofilm imaging.

## Supporting information

S1 Table Summary of specific Raman band assignment found for the three different species in planktonic and biofilm samples. Raman shift needs to be considered. Peak assignment based on Berger et al 2003, Carey et al 2017, Jung et al 2014 and Sil et al 2017.
(TIF)

S1 Fig Averaged processed Raman signal from mono-species biofilm cell for three different subgingival species. The bold line shows the average spectra of the mono-species biofilm cell. Dotted line is the averaged calibration spectra that is used for the prediction. *A*.*naeslundii* (An), *S*.*mutans* (Sm) and *V*.*dispar* (Vd).
(TIF)

## References

[1] Tanner A, Kent R, Maiden MFJ, Taubman MA. Clinical, microbiological and immunological profile of healthy, gingivitis and putative active periodontal subjects. J Periodontal Res. 1996;31: 195–204. 10.1111/j.1600-0765.1996.tb00484.x 8814590

[2] Abusleme L, Dupuy AK, Dutzan N, Silva N, Burleson JA, Strausbaugh LD, et al The subgingival microbiome in health and periodontitis and its relationship with community biomass and inflammation. ISME J. 2013;7: 1016–1025. 10.1038/ismej.2012.174 23303375 PMC3635234

[3] Socransky S s., Haffajee A d., Cugini M a., Smith C, Kent RL. Microbial complexes in subgingival plaque. J Clin Periodontol. 1998;25: 134–144. 10.1111/j.1600-051x.1998.tb02419.x 9495612

[4] Shi B, Chang M, Martin J, Mitreva M, Lux R, Klokkevold P, et al Dynamic Changes in the Subgingival Microbiome and Their Potential for Diagnosis and Prognosis of Periodontitis. mBio. 2015;6: e01926–14. 10.1128/mBio.01926-14 25691586 PMC4337560

[5] Ximenez-Fyvie LA, Haffajee AD, Socransky SS. Comparison of the microbiota of supra- and subgingival plaque in health and periodontitis. J Clin Periodontol. 2000;27: 648–657. 10.1034/j.1600-051x.2000.027009648.x 10983598

[6] Guggenheim B, Gmür R, Galicia JC, Stathopoulou PG, Benakanakere MR, Meier A, et al In vitro modeling of host-parasite interactions: the “subgingival” biofilm challenge of primary human epithelial cells. BMC Microbiol. 2009;9: 280 10.1186/1471-2180-9-280 20043840 PMC2818713

[7] Zijnge V, Ammann T, Thurnheer T, Gmür R. Subgingival Biofilm Structure. 2012 10.1159/000329667 22142954

[8] Xiao J, Hara AT, Kim D, Zero DT, Koo H, Hwang G. Biofilm three-dimensional architecture influences in situ pH distribution pattern on the human enamel surface. Int J Oral Sci. 2017;9: 74–79. 10.1038/ijos.2017.8 28452377 PMC5518976

[9] Thurnheer T, Karygianni L, Flury M, Belibasakis GN. Fusobacterium Species and Subspecies Differentially Affect the Composition and Architecture of Supra- and Subgingival Biofilms Models. Front Microbiol. 2019;10: 1716 10.3389/fmicb.2019.01716 31417514 PMC6683768

[10] Kommerein N, Stumpp SN, Müsken M, Ehlert N, Winkel A, Häussler S, et al An oral multispecies biofilm model for high content screening applications. PLOS ONE. 2017;12: e0173973 10.1371/journal.pone.0173973 28296966 PMC5352027

[11] Pantanella F, Valenti P, Natalizi T, Passeri D, Berlutti F. Analytical techniques to study microbial biofilm on abiotic surfaces: pros and cons of the main techniques currently in use.: 12. 10.7416/ai.2013.1904 23435778

[12] Rzhevskii A. The Recent Advances in Raman Microscopy and Imaging Techniques for Biosensors. Biosensors. 2019;9: 25 10.3390/bios9010025 30759840 PMC6468448

[13] Chao Y, Zhang T. Surface-enhanced Raman scattering (SERS) revealing chemical variation during biofilm formation: from initial attachment to mature biofilm. Anal Bioanal Chem. 2012;404: 1465–1475. 10.1007/s00216-012-6225-y 22820905 PMC3426672

[14] Gualerzi A, Niada S, Giannasi C, Picciolini S, Morasso C, Vanna R, et al Raman spectroscopy uncovers biochemical tissue-related features of extracellular vesicles from mesenchymal stromal cells. Sci Rep. 2017;7: 9820 10.1038/s41598-017-10448-1 28852131 PMC5575260

[15] Cals FLJ, Bakker Schut TC, Caspers PJ, Baatenburg de Jong RJ, Koljenović S, Puppels GJ. Raman spectroscopic analysis of the molecular composition of oral cavity squamous cell carcinoma and healthy tongue tissue. The Analyst. 2018;143: 4090–4102. 10.1039/c7an02106b 30083685

[16] Rebrošová K, Šiler M, Samek O, Růžička F, Bernatová S, Holá V, et al Rapid identification of staphylococci by Raman spectroscopy. Sci Rep. 2017;7: 14846 10.1038/s41598-017-13940-w 29093473 PMC5665888

[17] Strola SA, Schultz E, Allier CP, DesRoches B, Lemmonier J, Dinten J-M. Raman microspectrometer combined with scattering microscopy and lensless imaging for bacteria identification. In: Mahadevan-Jansen A, Vo-Dinh T, Grundfest WS, editors. San Francisco, California, USA; 2013 p. 85720X 10.1117/12.2002301

[18] Sil S, Mukherjee R, Kumar NS, S. A, Kingston J, Singh UK. Detection and classification of Bacteria using Raman Spectroscopy Combined with Multivariate Analysis. Def Life Sci J. 2017;2: 435 10.14429/dlsj.2.12275

[19] Tewes T. Entwicklung einer Methode zur Identifizierung von Mikroorganismen über Raman-Spektroskopie. Hochschule Rhein-Waal. 2019.

[20] Colniță A, Dina N, Leopold N, Vodnar D, Bogdan D, Porav S, et al Characterization and Discrimination of Gram-Positive Bacteria Using Raman Spectroscopy with the Aid of Principal Component Analysis. Nanomaterials. 2017;7: 248 10.3390/nano7090248 28862655 PMC5618359

[21] Stöckel S, Kirchhoff J, Neugebauer U, Rösch P, Popp J. The application of Raman spectroscopy for the detection and identification of microorganisms: Raman spectroscopy for microorganism detection and identification. J Raman Spectrosc. 2016;47: 89–109. 10.1002/jrs.4844

[22] Beier BD, Quivey RG, Berger AJ. Raman microspectroscopy for species identification and mapping within bacterial biofilms. AMB Express. 2012;2: 35 10.1186/2191-0855-2-35 22794329 PMC3599146

[23] Beier BD, Quivey RG, Berger AJ. Identification of different bacterial species in biofilms using confocal Raman microscopy. J Biomed Opt. 2010;15: 066001 10.1117/1.3505010 21198175 PMC3014224

[24] Zhu Q, Quivey RG, Berger AJ. Measurement of bacterial concentration fractions in polymicrobial mixtures by Raman microspectroscopy. J Biomed Opt. 2004;9: 1182 10.1117/1.1803844 15568938

[25] Cepeda-Pérez E, Moreno-Hernández C, López-Luke T, Monzón-Hernández D, de la Rosa E. Evaluation of bacterial presence in the root canal by Raman spectroscopy: a preliminary study. Biomed Phys Eng Express. 2016;2: 065006 10.1088/2057-1976/2/6/065006

[26] Carey PR, Gibson BR, Gibson JF, Greenberg ME, Heidari-Torkabadi H, Pusztai-Carey M, et al Defining Molecular Details of the Chemistry of Biofilm Formation by Raman Microspectroscopy. Biochemistry. 2017;56: 2247–2250. 10.1021/acs.biochem.7b00116 28418636

[27] Pätzold R, Keuntje M, Anders-von Ahlften A. A new approach to non-destructive analysis of biofilms by confocal Raman microscopy. Anal Bioanal Chem. 2006;386: 286–292. 10.1007/s00216-006-0663-3 16868726

[28] Beier BD, Quivey RG, Berger AJ. Confocal Raman microscopy for identification of bacterial species in biofilms. In: Miller BL, Fauchet PM, editors. San Francisco, California, USA; 2011 p. 78880D 10.1117/12.871819

[29] Keleştemur Seda, Avci Ertug, Çulha Mustafa. Raman and Surface-Enhanced Raman Scattering for Biofilm Characterization. Chemosensors. 2018;6: 5 10.3390/chemosensors6010005

[30] Keleştemur S, Çobandede Z, Çulha M. Biofilm formation of clinically important microorganisms on 2D and 3D poly (methyl methacrylate) substrates: A surface-enhanced Raman scattering study. Colloids Surf B Biointerfaces. 2020;188: 110765 10.1016/j.colsurfb.2019.110765 31955016

[31] Berger AJ, Zhu Q. Identification of oral bacteria by raman microspectroscopy. J Mod Opt. 2003;50: 2375–2380. 10.1080/09500340308233569

[32] Almarashi JFM, Kapel N, Wilkinson TS, Telle HH. Raman Spectroscopy of Bacterial Species and Strains Cultivated under Reproducible Conditions. Spectrosc Int J. 2012;27: 361–365. 10.1155/2012/540490

[33] Jung GB, Nam SW, Choi S, Lee G-J, Park H-K. Evaluation of antibiotic effects on Pseudomonas aeruginosa biofilm using Raman spectroscopy and multivariate analysis. Biomed Opt Express. 2014;5: 3238 10.1364/BOE.5.003238 25401035 PMC4230853

[34] Lu X, Al-Qadiri HM, Lin M, Rasco BA. Application of Mid-infrared and Raman Spectroscopy to the Study of Bacteria. Food Bioprocess Technol. 2011;4: 919–935. 10.1007/s11947-011-0516-8

[35] Feng Z, Hou J, Yu Y, Wu W, Deng Y, Wang X, et al Dissecting the Metabolic Phenotype of the Antihypertensive Effects of Five Uncaria Species on Spontaneously Hypertensive Rats. Front Pharmacol. 2019;10: 845 10.3389/fphar.2019.00845 31417403 PMC6682664

[36] El Senousy AS, Farag MA, Al-Mahdy DA, Wessjohann LA. Developmental changes in leaf phenolics composition from three artichoke cvs. (Cynara scolymus) as determined via UHPLC–MS and chemometrics. Phytochemistry. 2014;108: 67–76. 10.1016/j.phytochem.2014.09.004 25301664

[37] Fanesi A, Zegeye A, Mustin C, Cébron A. Soil Particles and Phenanthrene Interact in Defining the Metabolic Profile of Pseudomonas putida G7: A Vibrational Spectroscopy Approach. Front Microbiol. 2018;9: 2999 10.3389/fmicb.2018.02999 30564224 PMC6288191

[38] Villa JEL, Quiñones NR, Fantinatti-Garboggini F, Poppi RJ. Fast discrimination of bacteria using a filter paper–based SERS platform and PLS-DA with uncertainty estimation. Anal Bioanal Chem. 2019;411: 705–713. 10.1007/s00216-018-1485-9 30450510

[39] Guggenheim B, Giertsen E, Schüpbach P, Shapiro S. Validation of an in vitro Biofilm Model of Supragingival Plaque. J Dent Res. 2001;80: 363–370. 10.1177/00220345010800011201 11269730

[40] Gmür R, Guggenheim B. Antigenic heterogeneity of Bacteroides intermedius as recognized by monoclonal antibodies. Infect Immun. 1983;42: 459–470. 10.1128/IAI.42.2.459-470.1983 6196291 PMC264452

[41] De Gelder J, De Gussem K, Vandenabeele P, Moens L. Reference database of Raman spectra of biological molecules. J Raman Spectrosc. 2007;38: 1133–1147. 10.1002/jrs.1734

[42] Kumar S, Verma T, Mukherjee R, Ariese F, Somasundaram K, Umapathy S. Raman and infra-red microspectroscopy: towards quantitative evaluation for clinical research by ratiometric analysis. Chem Soc Rev. 2016;45: 1879–1900. 10.1039/c5cs00540j 26497386

[43] Huang R, Li M, Gregory RL. Bacterial interactions in dental biofilm. Virulence. 2011;2: 435–444. 10.4161/viru.2.5.16140 21778817 PMC3322631

[44] McCabe RM, Donkersloot JA. Adherence of Veillonella Species Mediated by Extracellular Glucosyltransferase from Streptococcus salivarius. Infect Immun. 1977;18: 726–734. 10.1128/IAI.18.3.726-734.1977 591064 PMC421295

[45] Vacca-Smith A., Bowen W. Binding properties of streptococcal glucosyltransferases for hydroxyapatite, saliva-coated hydroxyapatite, and bacterial surfaces. Arch Oral Biol. 1998;43: 103–110. 10.1016/s0003-9969(97)00111-8 9602288

[46] Koo H, Xiao J, Klein MI, Jeon JG. Exopolysaccharides Produced by Streptococcus mutans Glucosyltransferases Modulate the Establishment of Microcolonies within Multispecies Biofilms. J Bacteriol. 2010;192: 3024–3032. 10.1128/JB.01649-09 20233920 PMC2901689

