# Supplementary figures and images for: Confocal Raman microscopy to identify bacteria in oral subgingival biofilm models
Source: PLoS One. 2020 May 11;15(5):e0232912. doi: 10.1371/journal.pone.0232912 (PMC7213720; doi:10.1371/journal.pone.0232912)

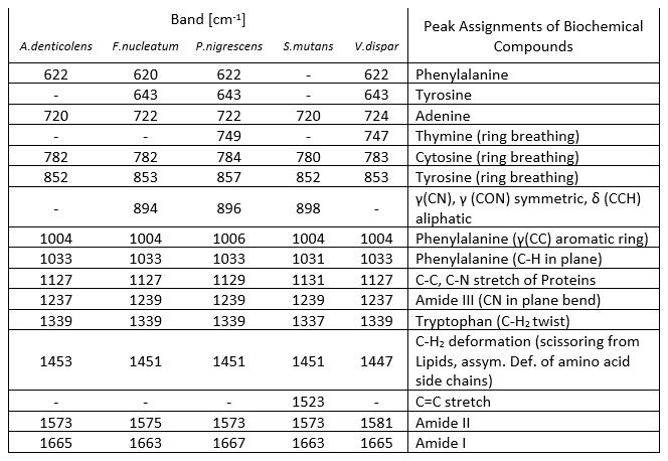

Supplement: S1 Table — Raman shift needs to be considered. Peak assignment based on Berger et al 2003, Carey et al 2017, Jung et al 2014 and Sil et al 2017. (TIF) [file pone.0232912.s001.tif]
